# GPx8 Expression in Rat Oocytes, Embryos, and Female Genital Organs During Preimplantation Period of Pregnancy

**DOI:** 10.3390/ijms21176313

**Published:** 2020-08-31

**Authors:** Jozef Mihalik, Andrea Kreheľová, Veronika Kovaříková, Peter Solár, Iveta Domoráková, Andriana Pavliuk-Karachevtseva, Alena Hladová, Silvia Rybárová, Ingrid Hodorová

**Affiliations:** 1 Department of Anatomy, Medical Faculty, Šafárik University, Šrobárova 2, 041 83 Košice, Slovakia; jozef.mihalik@upjs.sk (J.M.); andrea.krehelova@student.upjs.sk (A.K.); andriana.pavliuk-karachevtseva@upjs.sk (A.P.-K.); alena.hladova@student.upjs.sk (A.H.); silvia.rybarova@upjs.sk (S.R.); 2 Institute of Animal Physiology, Slovak Academy of Sciences, Šoltésovej 4-6, 040 01 Košice, Slovakia; kovarikova@saske.sk; 3 Department of Medical Biology, Medical Faculty, Šafárik University, tr. SNP 1, 040 11 Košice, Slovakia; peter.solar@upjs.sk; 4 Department of Histology and Embryology, Medical Faculty, Šafárik University, Šrobárova 2, 041 83 Košice, Slovakia; iveta.domorakova@upjs.sk

**Keywords:** glutathione peroxidase, antioxidant enzyme, immunofluorescence, immunohistochemistry, Western blot

## Abstract

This study aimed to detect the presence of glutathione peroxidase 8 (GPx8) in rat during preimplantation period of pregnancy. Females were killed on first (D1), third (D3), and fifth (D5) day of pregnancy. The presence of GPx8 in embryos was detected under the confocal microscope, the presence of GPx8 in genital organs was confirmed immunohistochemically, and the amount of GPx8 was determined using densitometry. We found that GPx8 is dispersed in the cytoplasm of oocytes, while after fertilization, it is concentrated in granules. From 4-cell stage till blastocyst, GPx8 reaction was found in the perinuclear region. In the ovary, GPx8 was seen in granulosa-lutein cells, in plasma of blood vessels, and inside Graafian follicles. In oviduct, GPx8 was detected in the plasma and in the extracellular matrix (ECM). Moreover, epithelial cells of isthmus were positive. In uterus, GPx8 was observed in the uterine glands, in the plasma, and in ECM. On D5, the enzyme disappeared from the uterine glands and appeared in fibroblasts. Densitometry revealed that the highest amount of GPx8 was on D1 and subsequently declined. To our knowledge, this is the first paper describing GPx8 presence in the oocytes, preimplantation embryos, and female genital organs in mammals. Our results improve the understanding of antioxidant enzymes presence during pregnancy in defense against oxidative stress, which is considered to be one of the main causes of infertility.

## 1. Introduction

Infertility, which is defined as the inability to achieve pregnancy after one year of regular unprotected sexual intercourse [1], became a significant worldwide public health problem [2]. The global average prevalence of infertility is estimated as 9% of reproductive-aged couples [3]. Current evidence links oxidative stress (OS) to male infertility, reduced sperm motility, sperm DNA damage, and increased risk of recurrent abortions and genetic diseases due to the large amounts of unsaturated fatty acids found in sperm cell membranes, which are highly susceptible to the harmful effects of reactive oxygen species (ROS) [4]. Many studies have also linked the prevalence of female infertility with an increase in oxidative stress levels in the various critical micro- or macro-environments in the body. Studies have shown that OS causes the abnormal expression of several proteins, which could lead to the pathophysiology of female infertility [5]. Although ROS are necessary for the normal course of the reproductive process, such as sperm capacitation, acrosome reaction, or sperm-oocyte fusion, OS is detrimental to fertility, pregnancy, and genetic status of the newborns [6].

OS is the result of the overproduction of ROS that overwhelms the antioxidant defense systems of the organism. Enzymatic antioxidants, such as glutathione peroxidases (GPxs) or superoxide dismutases (SODs), as well as nonenzymatic antioxidants (vitamin A, vitamin E, zinc, and selenium), are essential for maintaining adequate levels of ROS in the cell by disposing and removing excess free radicals. Any disruption in the ROS/antioxidants balance leads to a state of OS in the cell with damaging consequences [7].

The mammalian cell possesses multiple antioxidant systems to protect against ROS toxicity. For example, SODs are responsible for the conversion of superoxide anion to hydrogen peroxide, which in turn could be eliminated by several kinds of enzymes, such as catalase (CAT) or glutathione peroxidases (GPxs) [8]. The family of GPXs includes members that may contain either the rare amino acid selenocysteine (Sec) or the more common cysteine (Cys) [9]. The Sec subfamily in humans comprises four tetrameric GPxs (GPx1, GPx2, GPx3, and GPx6) and one monomeric peroxidase, GPx4. The Cys subfamily consists of three members. Two of them, GPx7 and GPx8, are monomeric proteins [10,11], and the last one, GPx5, is a tetrameric enzyme [12]. GPx7 and GPx8 are very similar, and evolutionary studies suggest that they both are derived from GPx4 [13]. GPxs use glutathione (GSH) to reduce hydrogen peroxide (H_2_O_2_) and other small hydroperoxides (ROOH) to water and corresponding alcohol. Each GPxs isoform has unique features concerning their subcellular localization, tissue distribution, substrate specificity, and their apparent biological function [14], e.g., GPx4 and GPx5 play roles in male fertility. At the same time, GPx7 and GPx8 are probably involved in correct protein folding and prevention of H_2_O_2_ leakage from endoplasmic reticulum to the cytosol [15]. Although the prevention of endoplasmic reticulum stress is considered to be a major function of GPx8, its some other unknown characteristics come to the light. Experiments on cancer HeLa cell line revealed, that GPx8 expression is induced by hypoxia [16], that silencing of GPx8 impacts membrane lipid composition [17], and that GPx8 level modulates endoplasmic reticulum Ca^2+^ concentration and fluxes [18]. Nevertheless, these results obtained from pathologically changed cells need to be verified on healthy cells that these mechanisms work also under physiological conditions.

The aim of our work is to determine the presence of antioxidant enzymes in rat experimental model during the earliest stage of pregnancy, since the oxidative stress is considered to be one of the main factors of infertility. The lack of information about whether individual glutathione peroxidases are present directly in mammalian oocytes and embryos during the preimplantation period of pregnancy prompted us to start this investigation. We decided to begin our work with GPx8, the latest discovered member of GPxs family. Moreover, to find possible interactions between mother and new individuals in protein expression, an investigation of GPx8 presence and its amount in female genital organs was also included in our study.

## 2. Results

### 2.1. Oocytes/Preimplantation Embryo Isolation and GPx8 IF Detection

The average numbers of isolated oocytes or female preimplantation embryos, reported as the mean ± S.D., were 10.4 ± 4.34 per female on D1, 10.5 ± 4.43 per female on D3, and 9.33 ± 3.79 per female on D5. On D1, only oocytes and zygotes, on D3 only 2-cell and 4-cell embryos, and D5 only blastocysts were considered as healthy, while other developmental types of preimplantation embryos were considered as degenerated. One half of all isolated oocytes/preimplantation embryos were employed for GPx4 detection (data not published here, work in progress), the second half was used for GPx8 detection.

**Figure 1:**
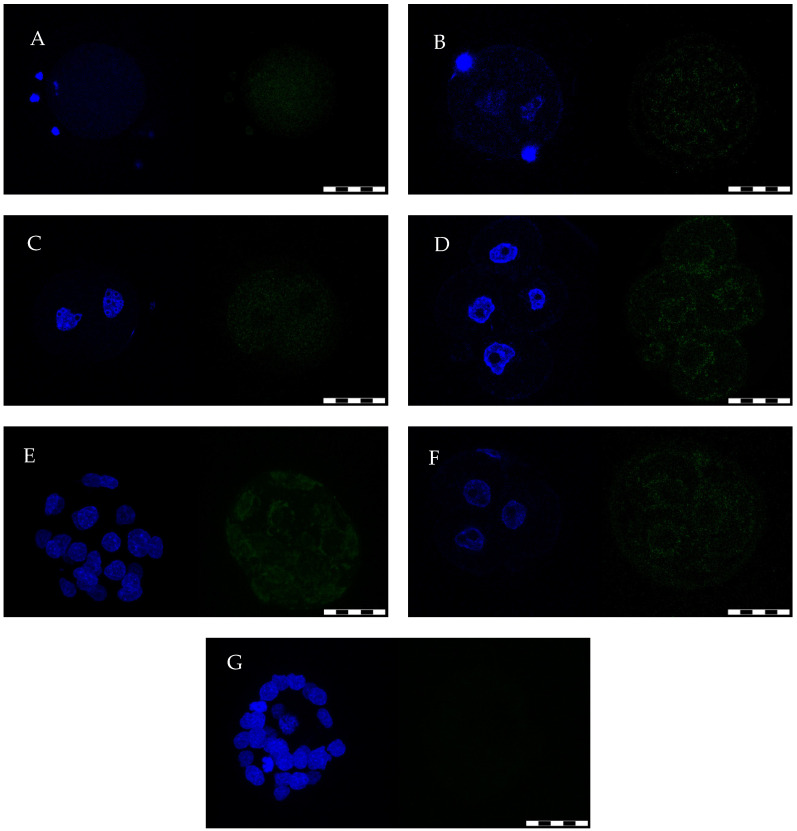
GPx8 presence in rat oocytes and preimplantation embryos detected under a confocal microscope. The left part of each picture shows nuclear material identified by Hoechst 33342; the right part of each picture shows the enzyme identified by an anti-GPx8 antibody conjugated with fluorescein isothiocyanate (FITC). Protein in unfertilized oocytes (**A**) was dispersed homogeneously in cytoplasm while in zygotes (**B**) and 2-cell embryos (**C**), it concentrated into many granules. Moreover, the protein was also detected in the cytoplasm of corona radiata cells; three of them are still attached to the left side of the oocyte (**A**). On the other hand, the protein was not detected in the head of sperms, two of which are attached to opposite sides of the zygote’s surface (**B**) but are blurred due to the different focusing positions compared to zygote’s pronuclei. Protein from 4-cell embryonic stage (**D**) till blastocyst (**E**) and even in the degenerated embryo (**F**) was predominantly observed in the area surrounding nucleus of each blastomere. Degenerated embryo here is represented by a 3-cell embryo isolated on D5 from the uterus, where the head of sperm is still attached to the upper surface of the zona pellucida. Control embryo (**G**) represents blastocyst on D5 after omitting antibodies against GPx8. Original magnification: ×400. Scale bar: 50 µm.

GPx8 enzyme was present in all examined oocytes/preimplantation embryos. This protein was dispersed homogeneously in the cytoplasm of unfertilized oocytes and granular membrane cells (Figure 1A), while after fertilization, it was concentrated in fine granules (Figure 1B). The same pattern was observed in 2-cell embryos (Figure 1C). From the 4-cell embryonic stage (Figure 1D) to blastocyst (Figure 1E), GPx8 reaction was found in the perinuclear region of all cells, and a weak reaction was also observed near the cytoplasmic membrane. The same pattern of protein appearance was detected in degenerated embryos (Figure 1F, here 3-cell embryo on D5).

### 2.2. Immunohistochemical GPx8 Detection

**Figure 2:**
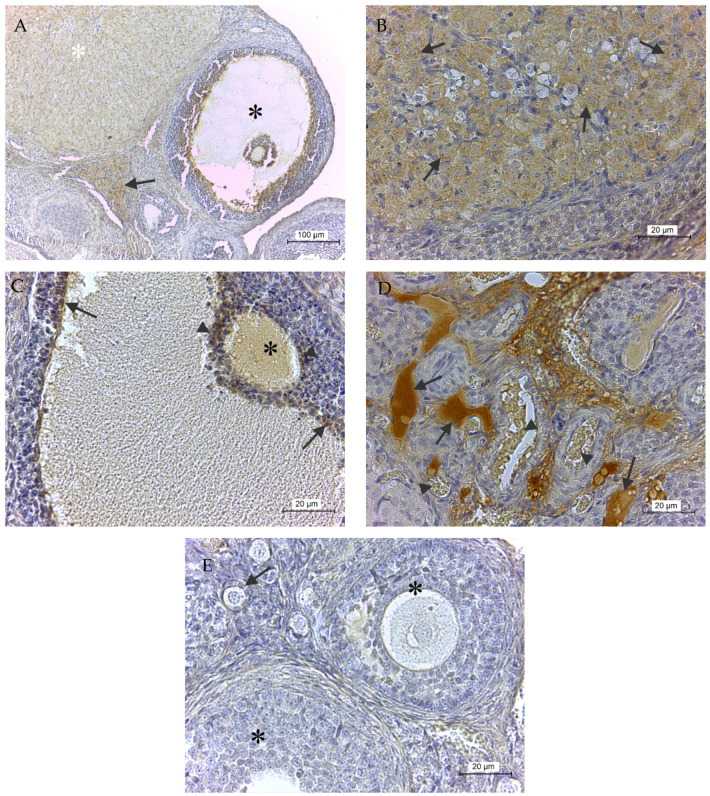
GPx8 presence in rat ovary. (**A**) shows an overall view of protein presence, where white asterisk means corpus luteum, the black asterisk means atretic Graafian follicle, and arrow means ovarian stroma. In detail, the protein was detected in granulosa-lutein cells (arrows) of corpus luteum (**B**), in the cytoplasm of the oocyte (black asterisk), in cells of corona radiata (arrowheads), in the innermost layer of granular cells (arrows) of atretic Graafian follicle (**C**), and in plasma in blood vessels (arrows) but not in erythrocytes (arrowheads) in (**D**). The enzyme was never detected in primary (arrow) and secondary follicles (black asterisk,) in (**E**).

The overall enzyme presence in the ovary is shown in Figure 2A. In detail, GPx8 was observed predominantly in the granulosa-lutein cells of corpus luteum (Figure 2A,B), in the cytoplasm of the oocyte, and in the innermost layer of granulosa membrane cells and corona radiata cells in atretic Graafian follicle (Figure 2A,C). The remaining plasma in the lumen of blood vessels and extracellular matrix of a perivascular connective tissue were GPx8 positive as well (Figure 2A,D). On the other hand, the protein has not been detected in primary or secondary follicles (Figure 2E).

**Figure 3:**
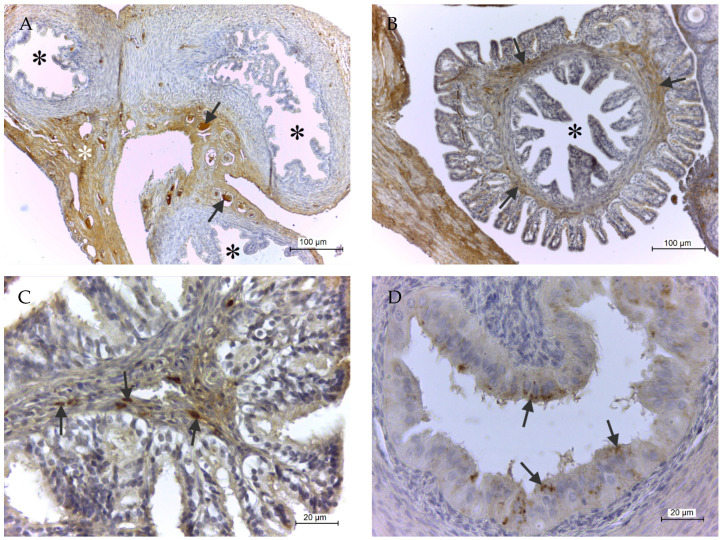
GPx8 presence in rat oviduct. (**A**) represents the overall view of protein presence, where the enzyme was predominantly seen in plasma in blood vessels (arrows) and extracellular matrix of fibrous tissue under the serous membrane (white asterisk). (**B**) shows protein presence in the infundibulum of the uterine tube (arrows). Black asterisks in (**A**,**B**) represent tubal lumen. (**C**) shows in detail that the enzyme in infundibulum was located in spindle-shaped fibroblasts (arrows). (**D**) shows the isthmic part of the uterine tube where the enzyme was present as granules in epithelial

Figure 3A provides an overall view of GPx8 localization in the Fallopian tube. The enzyme was present especially in plasma inside the blood vessels and in the extracellular matrix (ECM) of connective tissue in the serous membrane. Moreover, in the infundibulum of the uterine tube, spindle-shaped fibroblasts in the fimbriae pointing into the peritoneal cavity were also positive but not in mucosal folds pointing into the lumen of the salpinx (Figure 3B,C). Additionally, epithelial cells of the isthmus, but not of the ampulla, contained GPx8 positive granules on D1 and D3 (Figure 3D). GPx8 reaction was not observed in smooth muscle cells in any parts of salpinx (Figure 3A).

**Figure 4:**
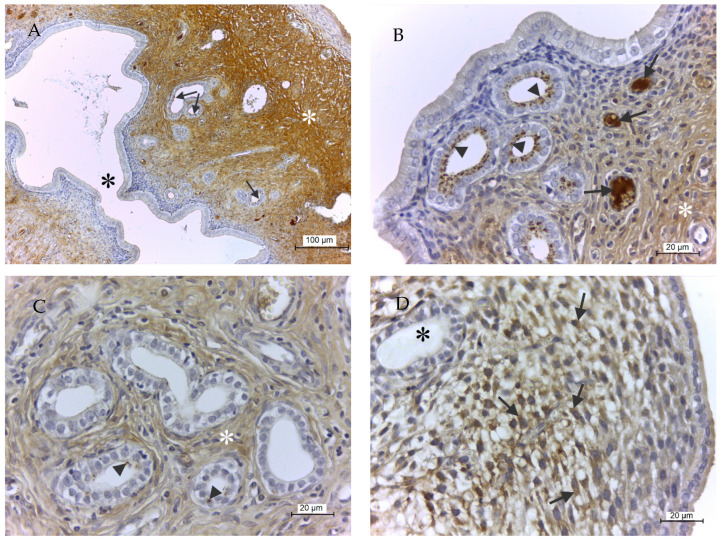
GPx8 presence in rat uterus. (**A**) is an overall view of protein presence on D1. The black asterisk represents the uterine cavity, the white asterisk means protein in endometrium and myometrium, and arrows indicate protein in uterine glands. (**B**) shows in detail that the enzyme was particularly detected in apical parts of cells of uterine glands (arrowheads), plasma in blood vessels (arrows), and extracellular matrix of the endometrium (white asterisk). On D3 (**C**), only very small residues of enzyme remained in the uterine glands (arrowheads), while enzyme in extracellular matrix of endometrium was still present (white asterisk). On D5 (**D**), the enzyme completely disappeared from uterine glands (black asterisk), but on the other hand, enzyme emerged in stellate-like fibroblasts of the endometrium (arrows). GPx8 was never observed in epithelial or muscular cells of the uterine wall, regardless of days of pregnancy.

The uterus was another organ, besides the salpinx, where changes in GPx8 expression dependent on the day of pregnancy. Figure 4A demonstrates the overall enzyme presence in the uterus on D1. Protein was observed predominantly in the apical part of epithelial cells lining of the uterine glands, plasma inside blood vessels, and ECM of endometrium and myometrium (Figure 4A,B). The presence of GPx8 on D3 was almost identical, with one exception that only small residua of protein persisted in uterine glands (Figure 4C). On D5, the enzyme not only completely disappeared from the uterine glands but, in addition, emerged in stellate-like fibroblasts forming decidua of the endometrium (Figure 4D). An enzyme was never detected in the epithelial lining of endometrium.

### 2.3. WB Analysis

The main band of GPx8 enzyme was detected at approximately 57 kDa in all examined organs and all examined days of pregnancy. Unlike ovary, in salpinx and uterus, an additional two bands of higher size were detected.

**Figure 5:**
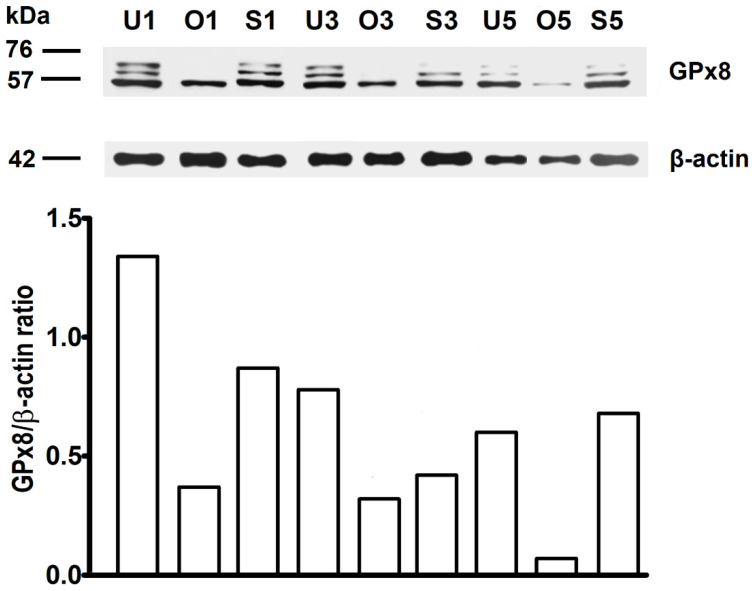
Western blot analysis of GPx8 amount in rat ovary (O), salpinx (S), and uterus (U). The number after the letter means the day of pregnancy. The main band of protein was detected at approximately 57 kDa in all examined organs. Moreover, in salpinx and uterus, two additional bands of higher size were detected. Densitometry, expressed as a ratio of GPx8 to β-actin, revealed that the highest amount of GPx8 enzyme was on D1 in the ovary (0.37), salpinx (0.87), and uterus (1.34). The amount of GPx8 in the ovary linearly decreased from D3 (0.32) to D5 (0.073) and similarly in the uterus (from 0.78 on D3 to 0.60 on D5). On the other hand, the lowest amount of protein in salpinx was observed on D3 (0.42), while it slightly increased on D5 (0.68).

Densitometry revealed that the highest amount of GPx8 enzyme was on D1 in ovary, salpinx, and uterus. On the other hand, the amount of GPx8 protein decreased linearly from D3 to D5 in ovary and uterus, while in salpinx, the lowest amount of protein on D3 slightly increased on D5. The statistical analysis of densitometry has not been evaluated, since pooled organs were used instead of individual organs (Figure 5).

## 3. Discussion

In our work, we found that enzyme GPx8 was present during the preimplantation period of pregnancy in all stages of new individuals, from unfertilized oocytes through zygotes to blastocysts. In addition, GPx8 was at the same time found in the ovary, uterine tube, and uterus of the mother. Moreover, the highest amounts of the protein in examined genital organs were on the first day of pregnancy, and then this amount declined.

Unique among the glutathione peroxidases family members, both GPx7 and GPx8 reside in the endoplasmic reticulum (ER). Unlike GPx7, which is entirely located inside the ER lumen, GPx8 is a transmembrane protein, with its active site facing the lumen [11,19]. The main source of ROS in the ER comes from intra-molecular disulfide bond formation during the maturation process of many secretory and membrane proteins. This process requires protein disulfide isomerase (PDI) and endoplasmic reticulum disulfide oxidase 1α (Ero1α) with subsequent H_2_O_2_ production [20,21]. However, such H_2_O_2_ cannot diffuse from ER to cytosol owing to the peroxidase activity of GPx8. This mechanism is essential to protect cells from Ero1α-mediated hyperoxidation and death [22]. Since embryos, during their development, produce large amounts of proteins needed for their growth and differentiation, it was not surprising that they contain also the GPx8.

According to our findings, GPx8 was observed in its typical localization around the nuclei, where the ER is situated, from the 4-cell stage to the blastocysts. Nevertheless, the protein was also detected in ovulated oocytes and zygotes up to the 2-cell stage. In these earlier stages of new individual development, GPx8 was dispersed in the cytoplasm. Thus, differences between maternal and embryonic GPx8 genome expression are evident. Briefly, oocytes accumulate a large set of proteins derived from the maternal genome. These maternal proteins are not only required for oocyte maturation and fertilization, but later, most of them are degraded, and their amino acid components are utilized for the synthesis of new proteins based on the embryonic genome [23]. In rodents, the start of embryonic gene transcription, called the zygotic genome activation (ZGA), accelerates in the transition from 2-cell to 4-cell stage, as opposed to the transition from 4-cell to 8-cell stage in humans [24]. Knowing differences in GPx8 distribution patterns, its enzymatic activity during maternal genome expression could be questioned since the enzyme was homogenously diffused in the cytoplasm. After fertilization, the enzyme even began to be concentrated in granules distant apart, and such protein arrangement resembles some kind of building framework. Indeed, this protein arrangement exists in GPx4, a vitally needed antioxidant enzyme, which is considered to be a direct ancestor of GPx8. GPx4, in mid-piece of mature spermatozoa, is a chemically inactive form and acts as a structural protein [25]. However, one can speculate that in related enzymes in a specific condition and specific cell types, similar arrangement could also be possible.

Of all known GPxs, only GPx3 and GPx5 have been described as extracellular enzymes so far. GPx3 is synthesized mainly by the kidney and released into the blood, where it is proposed to be a major scavenger of ROS because it accounts for nearly all of the glutathione peroxidase activity in plasma [26]. GPx3 from the plasma can also traverse the blood vessels and bind to basement membranes in many tissues [27]. GPx5 is released from epithelial cells into the epididymal lumen to protect maturing mammalian spermatozoa from OS [28]. Because GPx8 is anchored in the ER membrane, the presence of this enzyme in the blood plasma and also in perivascular connective tissue was surprising. The true role of this protein here is unclear, but one can hypothesize that GPx8 could be involved in the maintenance of protein disulfide isomerase (PDI), which in ER acts in protein folding, since GPx8 and GPx7 may accept PDI as a reductant more efficiently than does GSH [11]. Moreover, a recently published study suggested that PDI is present in human plasma at important levels and could be even used as a marker for some medical conditions [29].

On the other hand, ROS, and especially H_2_O_2_, are important regulators of endothelial cell homeostasis, which modulate their proliferation, migration, survival, and vasorelaxation [30]. Blood vessels are surrounded by extracellular matrix (ECM), which is an important microenvironmental component that modifies the kinetics of H_2_O_2_ consumption; therefore, it might be important during angiogenesis, endothelial cell migration and endothelial cell survival, and homeostasis regulation. This supports the existence of a crosstalk between ECM-dependent signaling and redox signaling to direct endothelial cell behavior [31]. ECM is a complex supramolecular material that includes collagens, elastin, proteoglycans, and glycosaminoglycans restricted to the basement membrane and interstitial spaces of all tissues [32]. ECM plays not only a role as a building network, but it is considered as an active structure in cell migration, division, and differentiation [33]. The ECM is degraded by matrix metalloproteinases (MMPs), which play important roles in tissue remodeling of female genital organs during cyclic changes, such as ovulation, menstruation, pregnancy, or cervical dilation during labor. Tissue inhibitors of metalloproteinases (TIMPs) act contrary to MMPs. ROS could trigger the tissue MMP activation and subsequent ECM degradation in the process of human trophoblast invasion [34] or rupture of fetal membranes, a major cause of preterm birth [35]. Previous studies have also indicated that MMP3, MMP-9, and TIMP-1 might participate in oviduct remodeling during the menstrual cycle [36], whereas MMP-8 activity participates in tissue remodeling processes during inflammation to establish successful Gonorrhea infection [37]. From this perspective, it is not surprising that we found GPx8, a member of the antioxidant enzyme family that degrades H_2_O_2_, in the ECM of the rat oviduct and uterus. Female genitals regularly undergo tissue remodeling during menstruation or pregnancy, so the balance between MMPs and TIMPs must also be maintained with the presence of antioxidant enzymes. Such equilibrium is essential for tissue stability because extensive and destructive degradation of the ECM could be seen in various pathological conditions, such as arthritis or cancer [38]. The only problem is that the GPx8 enzyme has not been described so far to be freely located in the ECM. We can assume that the real significance of the GPx8 presence in the plasma is not the H_2_O_2_ degradation or involvement in PDI maintenance in the bloodstream but the passage through the vessel walls into the ECM of the target organs. This is because we were not able to detect the presence of the enzyme in any cells immediately adjacent to the GPx8 detected in the ECM. Nevertheless, to prove or decline our working hypothesis, other types of experiments are needed.

The corpus luteum contains high levels of antioxidant enzymes, including SODs and GPxs, which protect luteal cells against ROS produced during steroidogenesis [39]. On the other hand, these oxygen radicals may also be functional in leading to luteolysis and apoptosis in corpus luteum during each reproductive cycle after prostaglandin F2 Alpha (PGF_2α_) stimulation [40]. Even exogenous hydrogen peroxide has been shown to inhibit progesterone synthesis in rat granulosa-lutein cells [41]. Since ROS are known to be involved in luteolysis [42], the corpus luteum requires antioxidant protection. It is known that when granulosa cells differentiate into corpus luteum, there is accompanying hypertrophy of the agranular endoplasmic reticulum, which seems to be further enhanced during pregnancy. This hypertrophy of the endoplasmic reticulum appears to reflect an increased demand for steroidogenesis and is also responsible for increased total protein observed in the pregnant corpus luteum [43,44]. Our finding that GPx8 is also present in granulosa lutein cells probably suggests that increased steroidogenesis in the corpus luteum needs protection against OS development.

From previous research, it is clear that some levels of H_2_O_2_ in the ovary are essential for ovulation to occur and for correct oocyte development [45]. The surge of LH, which is responsible for ovulation, also stimulates elevated ovarian ROS production [46]. On the other hand, the reduced reproductive outcome was recorded in oocytes retrieved from a follicular fluid (FF) exposed to higher H_2_O_2_ [47]. However, the limit between signaling and harmful H_2_O_2_ level is very small. It was estimated that 60 ng of ROS/oocyte is enough to maintain oocyte in diplotene arrest, whereas just a moderately increased ROS production of 80 ng causes meiosis resumption in oocytes [46]. Hence, it is not surprising that we detected GPx8 in Graafian follicles inside the ovary by immunohistochemistry and in ovulated oocytes and corona radiata cells by immunofluorescence in the oviduct. Probably, as the H_2_O_2_ production increases during follicular growth, demand for the presence of antioxidant enzymes involved in fine-tuning of the redox balance increases. Similarly, GPx1 protein was identified in bovine granulosa cells of large but not small healthy follicles, and the GPx1 gene expression was significantly higher in human cumulus cells from cumulus-oocyte complexes yielding a pregnancy [48].

The isthmus of the oviduct in mammals acts as a sperm reservoir, which is created by the binding of uncapacitated spermatozoa to the epithelial lining [49]. Such interactions increase the activities of antioxidant enzymes in spermatozoa, and sperm can survive here for up to several days [50]. Moreover, transcripts that encode GPxs enzymes were present in the mouse and human oviducts [51], but regional differences were observed. In cows, GPx3 [52] and GPx4 [53] were under-expressed in the ampulla and over-expressed in the isthmus. This is because the ampulla is the site of fertilization, and spermatozoa need ROS presence for the capacitation to occur, while spermatozoa in the isthmus need to be protected against ROS-induced damage as they constitute a sperm reservoir [54]. High GPxs activity was also detected in cow oviductal fluid. However, which GPxs would be expressed in the uterine fluid was unclear [52]. In our work, we detected GPx8 in the secretory cells of the isthmus. There are two possible reasons for the enzyme presence. First, that enzyme is released into the oviductal lumen, and second, the enzyme remains in the cells as the protection against OS, since secretory cells have intensive metabolism, and they are responsible for the secretion of many proteins and other factors which contribute to the formation of the oviductal fluid [55]. A similar situation was observed in cells of uterine glands that synthesize and secrete many substances, such as enzymes, growth factors, hormones, and transport proteins, collectively termed histotroph, into the uterine cavity, which subsequently influences blastocyst implantation and conceptus survival in mammals [56]. At this stage, the real role of the enzyme in different cells of the female genital system is unclear. However, at least in the secretory cells of the oviduct and in the cells of the uterine glands, the enzyme could be secreted into the lumen since we detected it as granules in the apical parts of the cells. On the other hand, in fibroblasts of endometrium and fimbriae of the Fallopian tube, the enzyme probably remains in the cells as the protection against OS since it was observed in the whole cytoplasm. In the rat uterus, the stimulus provided by the presence of embryo triggers the process of decidualization, when endometrial stromal fibroblasts proliferate and differentiate into decidual cells [57]. The decidua plays an essential role in protecting the embryo from being attacked by maternal immune cells and provides nutritional support for the developing embryo before placenta formation. The decidua also secretes many hormones, growth factors, and cytokines, such as prolactin, relaxin, or GnRH [58].

During the ovulation, the infundibulum covers the site of follicular rupture, and the fimbriae catch and conduct the oocytes into the oviductal lumen. One of the specific function of fimbriae is their chemotactic activity to estradiol levels from mature follicular fluid [59]. A limited number of studies have focused on the fibroblasts in the fimbriae; hence, it is not clear why GPx8 positive fibroblasts are situated in this part of the oviduct. Since fibroblasts synthesize and secrete the precursors of all the components of the ECM [60], the different components of the ECM in fimbriae, compared to other parts of the oviduct, might potentially influence their different motility and mucosal condition [61].

Concerning WB, we found that GPx8 is present in all observed organs. The main band of the protein was detected at approximately 57 kDa, and in salpinx and uterus, additional two bands of higher size were detected. This could indirectly prove that the enzyme is also located in sperm or components of seminal plasma, as these additional two bands have gradually disappeared with advancing pregnancy and were never observed in the ovary. Unfortunately, neither a sperm nor seminal plasma have not been analyzed in direct corroboration with our hypothesis. On the other hand, the actual band sizes for the GPx8 differ from predicted (24 kDa), probably due post-translational modification, such as phosphorylation or glycosylation, which both can increase the size of the proteins. The possibility, that protein forms dimers or trimers cannot also be excluded, as it was already proposed in hamster kidney with GPx [62]. Nevertheless, the nature of the protein and the molecular weight detected by Western blot will require a further demonstration of GPx8 by mass spectrometry.

Densitometry revealed that the highest amount of the enzyme was present in all organs on D1 of pregnancy when also ovulation occurs. On D3, in the amount of the protein decreased, and this decline continued in the ovary and uterus on D5 as well. On the other hand, in the salpinx, a successive mild increase of GPX8 amount was recorded on D5. Possible explanations for this phenomenon are that in the further course of pregnancy, higher ROS levels are necessary, or that other isoforms of GPXs or even CAT could replace GPx8 function.

## 4. Materials and Methods

### 4.1. Animals

All procedures involving animals adhered to the guidelines of the Committee for Ethical Control of Animal Experiments at Šafárik University, the American Psychological Association [63], and the Slovak State Veterinary and Alimentary Administration (permission no. Ro-11557/18-221/3, permission granted from 01/07/2018). All efforts were made to minimize both the number of animals and their suffering. Thirty female Sprague Dawley rats (320 g, 85–90 days old) were obtained from the Laboratory of Research Bio-models of the Šafárik University, and the experiments were performed in this animal facility as well. Organs from fifteen females were used for oocyte/embryo isolation and Western blot analysis. Organs from the other fifteen females were used for immunohistochemical detection. The animals were given free access to standard diet and water and were exposed to a 12 h light/12 h dark cycle. Females were mated with males of the same strain between 07:00 and 09:00 am. The presence of a vaginal plug was considered as a sign of successful mating. Such day was indicated as the first day of pregnancy (D1). Females were killed by a lethal dose of Zoletil (100 mg/kg; Virbac SA, France) on D1, D3, and D5.

### 4.2. Oocytes/Embryo Isolation and GPx8 Immunofluorescence (IF) Detection

Oviducts and uterine horns collected on each examined day from five females were placed on Petri dishes, and oocytes/preimplantation embryos were gently flushed out using phosphate buffer saline (PBS) + BSA (3 mg/mL) and carefully examined under the stereomicroscope. Subsequently, ovaries, oviducts, and uteri from corresponding females were pooled according to the day of pregnancy into Eppendorf tubes and stored at −80°C for Western blot analysis (see below).

Oocytes/preimplantation embryos were fixed in 4% paraformaldehyde (Merck, Darmstadt, Germany) in PBS at room temperature (RT) for 1 h and stored in 1% paraformaldehyde in PBS at 4 °C. Fixed oocytes/embryos were washed three times for 5 min in PBS containing 0.1% BSA (PBS/BSA) and transferred into PBS with 0.5% Triton X-100 (Sigma–Aldrich, Saint-Louis, MO, USA) for 1 h at RT. After permeabilization, oocytes/embryos were washed twice for 5 min in PBS/BSA and incubated in rabbit polyclonal anti-GPx8 antibody conjugated with FITC (#orb188222, 1:50, Biorbyt Ltd., Cambridge, United Kingdom) overnight at 4 °C. Next day, oocytes/embryos were washed six times for 5 min in PBS/BSA at RT, counterstained with Hoechst 33342 DNA staining (10 μL/mL in PBS; Sigma-Aldrich, Saint-Louis, MO, USA) for 5 min at RT, washed in one drop of PBS/BSA, and mounted on glass slides with Vectashield (Vector Laboratories, Burlingname, CA, USA). Oocytes/embryos were finally observed and photographed using a confocal laser scanning microscope (Leica TCS SPE, Leica, Mannheim, Germany) with 400× magnification.

### 4.3. Immunohistochemistry

Ovaries, oviducts, and uteri collected from five females on D1, D3, and D5 were embedded into blocks of paraffin using standard procedures. Sections of 5μm were cut, deparaffinized, and rinsed in EnVision Flex Wash Buffer (#K800721-2, Agilent Dako, Santa Clara, CA, USA, later in the text referred to as wash buffer). Endogenous peroxidase activity was blocked by incubation of slides in a mixture of methanol and hydrogen peroxide. After another rinsing in wash buffer, antigens were revitalized in the microwave. Slides were rinsed in wash buffer, and 2% milk blocking solution in Tris buffer was added. The primary anti-GPx8 rabbit polyclonal antibody (#orb183909, Biorbyt Ltd., Cambridge, United Kingdom) was applied overnight at 4 °C, followed by rinsing in wash buffer. Subsequently, Biotinylated Link (#K0675, Agilent Dako, Santa Clara, CA, USA) was used, then slides were rinsed with wash buffer, and Streptavidin-HRP (#K0675, Agilent Dako, Santa Clara, CA, USA) was applied. The tissue sections were again washed in wash buffer, and 3,3-diaminobenzidine (DAB) (#K5207, Agilent Dako, Santa Clara, CA, USA) was applied. The slides were rinsed in tap water, counterstained by hematoxylin, and embedded into Pertex. The negative controls were created by omitting the primary antibody. Two observers independently evaluated the results of the immunostaining under a light microscope.

### 4.4. Western Blot (WB) Analysis

The tissue was washed twice with ice-cold PBS and homogenized into the RIPA buffer (1xPBS, 1% Nonidet P-40, 0.5% sodium deoxycholate, 0.1% SDS, Thermo Fisher Scientific, Inc., Waltham, MA, USA) in the presence of protease and phosphatase inhibitor cocktail (Thermo Fisher Scientific, Inc.). Lysates were incubated for 45 min on ice and sonicated for 30 s at 30 V (Sonopuls HD 2070; Bandelin electronic GmbH & Co. KG, Berlin, Germany) to shear the DNA. After the centrifugation at 10,000× *g* for 10 min at 4 °C, the supernatant was transferred into a new microcentrifuge tube. Protein samples were separated on 12% SDS-polyacrylamide gel, electroblotted onto Immobilon-P transfer membrane (Millipore Co., Billerica, MA, USA), and detected using anti-GPX8 (#orb183909, 1:200; Biorbyt Ltd., Cambridge, UK) and anti-β-actin (clone AC-74, 1:10,000; Sigma-Aldrich) primary antibodies. The membranes were then incubated with secondary horseradish peroxidase-conjugated antibodies (Goat anti-Rabbit IgG F(AB’) 2 diluted at 1:10,000, PI-31461 and Goat anti-Mouse IgG F(AB’) 2 diluted at 1:10,000, PI-31436, Pierce, Rockford, IL, USA) for 1 h and the antibody reactivity was visualized with ECL Western blotting substrate (PI-32106, Pierce) using Kodak Biomax film (#1788207, Sigma-Aldrich, Saint-Louis, MO, USA). The films were scanned using GS-800 Calibrated Densitometer, and Image J software version 1.52 (NIH, National Institute of Health, Bethesda, MD, USA) was used for protein quantification.

## 5. Conclusions

In these experiments, we found that GPx8 is present in both oocytes and preimplantation embryos of rats. Moreover, the enzyme was also detected in different cells of ovary, salpinx, and uterus. The amount of GPx8 in all organs varied between days of pregnancy. The fact that the GPx8 was found in oocytes/embryos and concurrently in all genital organs of the mother during the entire preimplantation phase of pregnancy suggests that glutathione peroxidases are important for the early development of a new individual. In order to know the physiological effect of this enzyme on the course of the reproductive process, it would be suitable to perform experiments studying the knock out effect of GPx8 on the embryo development. It is questionable whether the deficiency of this enzyme would also affect the development of embryos or whether it would be overlaid with some possible compensatory mechanisms. So far, GPx4 is the only member of glutathione peroxidase family known as a vital enzyme necessary for the development of a new individual, since in its deficiency mice embryos die during second week of pregnancy and no other antioxidant enzyme can replace it. On the other hand, GPx4 is considered to be a direct ancestor of GPx8, so one can assume that GPx8 could also have vital properties, if its depletion could change membrane composition [17] and Ca^2+^ fluxes [18] also in healthy cells.

It would be worthwhile to verify the presence of this enzyme in human embryos and genital tissues, as oxidative stress is considered to be one of the main causes of human infertility and antioxidant enzymes play an important role in defense against it. In addition, only polyclonal GPx8 antibodies are currently available against rat experimental model, but monoclonal antibodies, which achieve more accurate results, are already available for human samples. This should be important mainly to confirm GPx8 presence in plasma and extracellular matrix.

To our knowledge, this is the first paper describing GPx8 presence in the oocytes, preimplantation embryos, and female genital organs in mammals.

## Abbreviations

BSA	Bovine serum albumin
CAT	Catalase
Cys	Cysteine
D1–D5	Corresponding day of pregnancy
ECM	Extracellular matrix
Ero1α	endoplasmic reticulum disulfide oxidase 1α
FF	Follicular fluid
FITC	fluorescein-5-isothiocyanate
GnRH	gonadotropin-releasing hormone
GPxs	Glutathione peroxidases
GPx8	Glutathione peroxidase 8
GSH	Glutathione
H_2_O_2_	hydrogen peroxide
IF	Immunofluorescence
IgG	Immunoglobulin G
OS	Oxidative stress
MMPs	Matrix metalloproteinases
PBS	Phosphate buffer saline
PGF_2α_	Prostaglandin F2 Alpha
PDI	protein disulfide isomerase
ROOH	Hydroperoxides
ROS	Reactive oxygen species
RT	Room temperature
Sec	Selenocysteine
SODs	Superoxide dismutases
TIMPs	Tissue inhibitors of metalloproteinases
WB	Western blot
ZGA	Zygotic genome activation
